# Relationship between Condylar and Ramal Asymmetries and ABO and Rh Blood Groups

**DOI:** 10.2174/0115734056378135250618150104

**Published:** 2025-06-24

**Authors:** Mehmet Emrah Polat, Halil Ibrahim Durmus, Mehmet Gul

**Affiliations:** 1 Department of Oral and Maxillofacial Surgery, Dentistry Faculty, Harran University, 63300 Sanliurfa, Turkey; £ Present Address: Department of Oral and Maxillofacial Surgery, Dentistry Faculty, Adiyaman University, 02030 Adiyaman, Turkey; 2 Department of Peirodontology, Dentistry Faculty, Harran University, 63300 Sanliurfa, Turkey; £ Present Address: Department of Periodontology, Dentistry Faculty, Nevsehir Haci Bektas Veli University, 50300, Nevsehir, Turkey

**Keywords:** Blood groups, Condylar asymmetry, Temporomandibular joint, Healthy population, Mandibular anatomical variations, Rh groups

## Abstract

**Objective:**

The association between ABO and Rh blood groups and diseases is an intriguing topic that continues to be studied, but their potential influence on mandibular asymmetry has not been explored. Temporomandibular joint (TMJ) disorders are multifactorial, and subtle anatomical variations may be linked to genetic predispositions. Our study aims to investigate the relationship between ABO and Rh blood groups and mandibular condylar and ramal asymmetries in a healthy adult Turkish population.

**Materials and Methods:**

This study included 149 adult patients (67 males, 82 females) who had no history of systemic diseases, craniofacial deformities, or TMJ-related complaints. Asymmetry was assessed in panoramic radiographic images using a formula developed in a previous study. The chi-square and Kruskal-Wallis tests were used to analyze differences among ABO groups while the Mann-Whitney U test was used for Rh groups.

**Results:**

No significant difference was found in terms of gender distribution, Rh factor or age between ABO or Rh groups. However, there was a significant difference in condylar asymmetry index (CAI) between ABO groups (p < 0.05). Pairwise comparisons revealed that individuals with AB blood type exhibited significantly higher CAI values compared to those with B blood type. No statistically significant differences in asymmetry indices were observed between Rh groups.

**Conclusion:**

The findings of our study indicate the existence of a significant relationship between blood groups and asymmetry indices in a healthy population. The significant differences in condylar asymmetry between AB and B blood groups indicate a possible association between blood type and mandibular anatomical variations, rather than a causal relationship. Further studies are needed to confirm these findings and to understand the underlying mechanisms of the relationship between blood groups and mandibular asymmetry.

## 1 INTRODUCTION

The etiology of temporomandibular disorders (TMDs) has been extensively investigated, revealing that factors, such as trauma, stress, arthritic changes resulting from systemic conditions, and parafunctional habits, can contribute to TMD symptoms. While craniofacial morphology plays a role in TMD, mandibular and facial asymmetries have also been observed in individuals without TMD symptoms. Emotional and psychological aspects may also influence the occurrence of TMD. In addition, the craniofacial morphology also plays a significant role [1].

Bilateral symmetry is defined as the equal distribution of parts on two sides of the body. Facial symmetry, encompassing various components, is defined as the overall harmony among these components. Conversely, asymmetry refers to the lack of this harmony [2]. Aesthetic changes in the face can significantly impact quality of life, being particularly important for young individuals, in whom appearance may directly influence social relationships [3]. Mandibular asymmetry directly influences facial appearance and can cause functional problems due to the mandible’s role in the stomatognathic system. Because condylar asymmetry is a primary cause of mandibular asymmetry, it holds great importance in terms of its impact on TMDs and facial asymmetry. Therefore, radiological evaluation of condylar asymmetry is an essential component of assessing TMD patients [4]. A method of quantifying vertical asymmetry during mandibular condylar and ramal assessment has been widely adopted by researchers [5].

Infection or failure of condylar cartilage has been reported to cause asymmetry, with endochondral ossification and bone attachment likely to be the primary growth factors. In addition, muscle activity also has an effect on facial asymmetry, given the differences in cases of unilateral crossbite [6]. Although studies have shown that ethnicity and gender differences have no effect on asymmetry perception, the relationship between ethnicity and gender variables and asymmetry has not been well investigated. Although racial differences have been found in dental anatomy and cranial morphology, the role of race in the pathogenesis of mandibular asymmetry has not been addressed [6].

The ABO blood grouping system categorizes blood into four groups: O, A, B, and AB. Group O produces antibodies against A and B antigens, while groups A and B have erythrocytes with A and B antigens, respectively, and produce antibodies against the opposite antigen. Group AB carries both A and B antigens, and does not form antibodies against either antigen [7].

Blood type, a hereditary factor, is associated with various diseases [8]. The Rhesus (Rh) blood grouping system classifies blood as Rh negative or positive based on the presence of the D antigen in red blood cells (RBCs). Both the ABO and Rh blood groups have been linked to diseases, including diabetes mellitus, cardiovascular disorders, periodontal diseases, infections, and cancer. RBCs play a crucial role in the inborn immune response, contributing to the emergence and maintenance of immunity [9, 10].

Several studies have investigated the relationship between ABO blood groups and various systemic diseases. Significant differences between ABO groups have been observed in studies on oral cancers, salivary gland tumors, pancreatic tumors, colitis, diabetes, and ischemic heart diseases [11]. Maxillofacial deformities can be acquired or inherited. It is clear that genetics plays an important role in inherited maxillofacial deformities. Facial development is multifactorial, and this multifactoriality can make it difficult to determine the genetic pattern of deformities. Nevertheless, epidemiologic evaluations may find associations between certain deformities and certain genetic traits and contribute to the literature in terms of diagnosis and treatment. One of the most important human genetic traits is the ABO and Rh blood group systems [12].

The clinical importance of ABO blood groups should extend beyond transfusion medicine, as the antigens of these blood groups are also expressed at very high rates on the surfaces of various cells and tissues, including epithelium, platelets and vascular endothelium [6]. For this reason, many studies have reported a relationship between these blood groups and dozens of diseases [13].

The effects on bone tissue were also observed and the relationships between blood groups and bone mineralization and osteoporosis were compared and it was reported that different blood groups had significant differences in terms of bone mineralization and osteoporosis risk and had an effect on bone metabolism [13, 14].

It is well documented in the literature that ABO blood groups significantly affect the concentrations of Factor VIII (FVIII) and von Willebrand factor (vWF) in plasma [4]. It has been shown in the literature that the FVIII-vWF complex has a direct effect on osteoclast activity and may play a role in bone resorption processes by inducing cellular apoptosis. It is stated that this mechanism is directly related to bone remodeling and possible bone loss [5]. In this context, interindividual differences in FVIII and vWF levels may affect the changes that may occur in the mandibular bone structure [15, 16].

Panoramic radiographs, which were used by Habets *et al*. [5], provide an index of the height difference between the right and left condyle. The vertical component of the structures visible on these radiographs is less subject to distortion in the posterior regions than in the anterior regions, making this method sufficiently accurate. The fact that panoramic radiographs do not require high radiation doses and complex equipment, as well as their widespread use in dental practice, makes them suitable for routine evaluation in the clinical setting [17].

To our knowledge, the literature has not previously specifically examined the potential impact of various blood groups, particularly ABO and Rh blood groups, on the TMJ and surrounding anatomical structures. While the relationship between systemic diseases and blood groups has been extensively studied, including conditions affecting other parts of the body, there is a significant gap in the literature regarding the role that these genetic factors may play in maxillofacial anatomy, particularly in relation to TMJ function and structural asymmetry. Given the complex and multifactorial nature of TMJ-related disorders, it is possible that blood groups contribute to subtle anatomical variations that affect joint symmetry and consequently have an indirect effect on facial appearance and function. Our study was planned as a pilot study to fill this gap in the literature by aiming to examine the relationship between ABO and Rh blood groups and condylar and ramal asymmetry indices. In this study, we aimed to conduct a detailed analysis in an adult Turkish population, focusing on individuals without TMJ disorders or facial asymmetry symptoms, and to establish a clear basis for understanding these relationships in a healthy cohort. Widely recognized and validated by Habets *et al*. for the assessment of vertical asymmetry in mandibular condylar and ramal height [5], this research will generate data that can serve as a basis for future studies in this area.

## 2 MATERIALS AND METHODS

This retrospective study was implemented to evaluate the demographic and radiographic data of patients at Harran University Faculty of Dentistry. The specific focus of this study was asymmetry index measurements on panoramic radiographs. Between April and May 2024, a retrospective screening was conducted on panoramic radiographs obtained from routine dental examinations of 149 patients without systemic diseases, craniofacial deformities, orthodontic treatment history, or TMJ-related complaints.

A post hoc power analysis was conducted using G*Power 3.1 software, which indicated a power level of 78.6% (Cohen’s f = 0.27, total sample size = 149). The inclusion and exclusion criteria for the study are presented in detail in Table **1**. The total study cohort included 67 male and 82 female patients, as seen in Tables ** 2** and **3**. In order to conduct an accurate and reliable study, only patients who had panoramic film records with radiograph quality that could be used for asymmetry index calculation and whose age, gender and blood group information were recorded in the patient files were included in the final analysis. Subjects with insufficient demographic information or unsuitable panoramic images for asymmetry index measurement were excluded from the study. The study was conducted in accordance with Harran University Clinical Research Ethics Committee (approval number: HRU/24.02.69). Thus, compliance with ethical norms was ensured.

Panoramic images were acquired using a PaX-I machine (Vatech Panoramic imaging machine, South Korea), with standardized settings of mA = 8, kVp = 60, and an exposure time of 10 seconds for consistent image quality. The images were evaluated using EasyDent software (version 4.1.5.9) that contains the parameters used in the calculation of asymmetry, such as calibration, measurement and angulation. To minimize distortions and magnification errors, all patients were positioned according to a standardized head positioning protocol, and the chin rest and bite block were used to stabilize head positioning and reduce movement artifacts during exposure. Additionally, the EasyDent software used for image evaluation allows for precise digital measurements, reducing the risk of human error associated with manual assessments.

To evaluate the anatomical structures needed for mandibular asymmetry measurements, the most distal points of the mandibular condylar process and mandibular ramus were identified and marked as points A and B, respectively, and after this, a straight reference line (X) was drawn between these points. In order to analyze the condylar height correctly, a perpendicular line (Y) was drawn from the uppermost point of the condyle on either side, intersecting the X line at point C, as illustrated in Fig. (**1**). Condyle height (CH) was measured as the distance between points C and A, and ramal height (RH) as the distance between points A and B. These measurements were consistently performed by the same investigator to avoid measurement variability. The measurements were also performed in a blinded manner, ensuring that the investigator was unaware of patient details to minimize potential bias. The calibration tool provided by EasyDent software was used to improve accuracy throughout the measurement process. To calculate mandibular condylar and ramal asymmetries, we used the Habets formula, which is based on radiographic data, widely used and accepted in the literature [5]. A detailed illustration of the measurement method is presented in Figs. (**1** and **2**).

Statistical analysis was performed using SPSS Statistics version 20.0 (IBM Corp., Armonk, NY, USA). Descriptive statistics were calculated, and the significance level was set at p < 0.05 for all tests. The normality of the condylar asymmetry index (CAI) and ramal asymmetry index (RAI) variables was tested using the Kolmogorov-Smirnov test. Chi-square tests were used to analyze the sex and Rh factor prevalence in ABO blood groups. The Kruskal-Wallis test was used to assess differences among ABO blood groups, while the Mann-Whitney U test was used for Rh groups. To control for the increased risk of Type I errors in multiple pairwise comparisons, Bonferroni correction was applied to the post hoc analyses following the Kruskal-Wallis test for ABO groups. This adjustment ensured that the significance threshold was appropriately corrected to maintain statistical validity.

## 3 RESULTS

The Ethics Committee of Harran University reviewed and granted approval to the study protocol and procedures with confirming adherence to the required ethical standards (approval number: HRU/24.02.69). The study sample consisted of 149 panoramic radiographs carefully selected from a total of 67 male and 82 female patients. These patients had no abnormalities associated with symmetry or the TMJ. The patient selection process was designed to minimize potential factors that could affect the asymmetry index.

CAI, RAI, and age variables were statistically analyzed to assess their distribution patterns by using the Kolmogorov-Smirnov test, which indicated that they did not follow a normal distribution and also the need for nonparametric statistical analyses to ensure the robustness and accuracy of the results.

Tables **2** and **3** provide a detailed breakdown of the descriptive statistics and the results of the statistical analyses for the patients in each group. Sex distribution, Rh distribution, and patient ages across the ABO groups were analyzed using chi-square and nonparametric tests, revealing no statistically significant differences among the groups. These tables not only summarize key demographic variables, but also highlight specific findings regarding asymmetry indices between different blood groups and indicate which statistical tests were used.

The distribution of gender, Rh factor and patient age among the various ABO blood groups was analyzed using the chi-square test for categorical variables and the nonparametric Kruskal wallis tests for numerical variables. The analyses showed that there were no statistically significant differences in these demographic variables between ABO blood groups, indicating a balanced distribution of these variables. However, comparisons of asymmetry indices between different ABO blood groups revealed significant differences in relation to CAI that reached statistical significance, suggesting that blood group may be an important factor in condylar asymmetry (p < 0.05). In particular, pairwise comparisons revealed that patients of blood group AB exhibited significantly higher CAI values compared to those of blood group B. In contrast to the results for ABO blood groups, analyses within Rh groups did not reveal a statistically significant difference in CAI or RAI.

## 4 DISCUSSION

Although several studies have evaluated the relationship between condylar asymmetry and occlusal asymmetry, TMJ diseases, and crossbite, the relationship between condylar asymmetry and the ABO and Rh blood groups has not previously been investigated [18]. Asymmetrical use can lead to asymmetrical mandibular development. In addition, asymmetries between the sides of the mandible may result from an adaptive response, leading to condylar remodeling [19].

The incidence of TMD is higher in individuals with asymmetries, indicating that asymmetry may be a significant factor in TMD development [20]. Mandibular asymmetry can be assessed using several different methods, including panoramic radiographic evaluation, which has frequently been used in previous studies [21]. Panoramic radiographs offer advantages, such as low cost and low radiation exposure, but they also have limitations due to magnification risks, distortion, and superimposition of anatomical structures [21, 22].

Cone-beam computed tomography is considered the gold standard for assessing mandibular asymmetries [20]. However, its disadvantages include limited use in routine dental imaging, high radiation dose, and high cost. Therefore, we utilized panoramic radiographs. This was the first study to investigate the relationship between the ABO and Rh blood groups and vertical condylar and ramal asymmetry using the method of Habets *et al*. [5]

Mandibular condylar asymmetry can affect both facial aesthetics and jaw function. Studies have shown that this condition may lead to chewing difficulties and can be associated with temporomandibular joint (TMJ) disorders. When condylar asymmetry is present, the lower jaw may shift slightly to one side, affecting facial symmetry and potentially influencing an individual's self-confidence. In more severe cases, this asymmetry can result in malocclusions that may require orthodontic treatment or even surgical intervention [23, 24].

Lundstrom [25] classified the possible causes of facial and dental arch asymmetries as genetic, environmental, or a combination of these factors. Autoimmune diseases and inflammatory conditions have also been associated with TMDs [26]. The ABO system is the most widely used blood grouping system. Other important blood grouping systems include the Rh and MN systems. The clinically significant and widely used ABO and Rh systems are determined based on the nature of different proteins found at the surfaces of RBCs [11].

The genes encoding ABO and Rh blood group antigens are located on chromosomes 9q34.2 and 1p36.11, respectively. Several studies have demonstrated a relationship between certain rheumatic and autoimmune diseases and blood groups. Two studies that evaluated pemphigus vulgaris, an autoimmune condition, found no significant relationship with ABO blood groups [27]. Glycoconjugate structures on RBCs have various functions, including roles as receptors, transporters, channels, structural proteins, adhesion molecules, and enzymes. Although the exact mechanisms explaining the relationships between blood group antigens and adhesion molecules are not fully understood, these may play a role in disease processes [28].

Such structures also play active roles in cell physiology and pathology. Despite the significant association between ABO blood groups and various diseases, there are controversies regarding resistance to certain diseases due to the absence of antigens in some groups. The presence or absence of these antigens is associated with health and disease. Such antigens are not only found in RBCs but also in leukocytes, platelets, plasma proteins, and enzymes. They can also be present in soluble form in body fluids such as sweat, saliva, amniotic fluid, and urine [29-32].

The presence or absence of A/B antigens affects host defense against infection. Although many studies have demonstrated a relationship between ABO blood groups and diseases, some researchers have published contradictory findings. Non-O blood groups are generally more susceptible to diseases compared to O blood groups [28].

The relationship between ABO blood groups and a variety of diseases warrants further investigation. A meta-analysis of 17 relevant studies reported a statistically significant difference between the AB group and other blood groups in terms of periodontitis [33]. However, there were no differences for gingivitis, and inconsistencies were noted among the studies. The authors emphasized the need for further studies with larger samples.

In a study by Kundu *et al.* [34], the incidence of aggressive periodontitis was higher in individuals with the AB blood group. We similarly found a higher rate of condylar asymmetry in such patients. We believe that the presence of both A and B antigens in the AB group may explain this outcome.

There may be a relationship between blood groups and periodontal and dental diseases. Individuals with AB type blood are less resistant to childhood caries [35, 36]. This finding corresponds with the higher rates of condylar asymmetry in the AB group observed in our study. However, further research using comprehensive and homogeneous study groups is needed to investigate the association between blood group types and diseases.

In our study, homogeneity was ensured by the balanced distribution of sexes and ages across the groups, which did not result in significant differences. We believe that our findings regarding the relationship between blood groups and condylar asymmetry address an unexplored area in the literature, potentially providing a foundation for future studies. The inconsistencies among studies may be due to varying methodologies as well as to geographical and genetic influences of blood groups in different populations [37].

In a study of oropharyngeal and oral cancer cases, a positive correlation was noted between blood groups A and AB and cancer incidence [38]. The authors attributed this to the immunological properties of cancer cell antigens, which resemble those of blood group A. In individuals with blood group O, these antigens may inhibit tumor growth, offering a protective mechanism. Individuals with blood types A and AB, who lack A antibodies, may be at greater risk for carcinomas.

When comparing the right- and left-sided CH and RH values across ABO and Rh blood groups, no differences were observed between the Rh groups. However, a statistically significant difference was found among the ABO groups, with significantly higher asymmetry scores in the AB group. The absence of differences between Rh groups and the similarity in sex, age, and Rh group distributions among the ABO groups indicate the homogeneity of our study population, enhancing the reliability of our comparisons.

ABO antigens are thought to be evolutionarily useful for conferring resistance to pathogens [39]. A and B antigens are mostly secreted by cells and are present in the human bloodstream. Groups that do not secrete antigens are considered at greater risk for various infections [40]. The observed higher asymmetry index in AB group patients may be associated with the presence of both A and B antigens; however, the biological mechanisms underlying this association are not yet understood. Future cellular and genetic studies are needed to explore whether ABO antigens influence condylar resorption. The lack of a significant difference in ramal asymmetry values may be explained by the absence of ramal resorption.

Numerous studies have documented the significant influence of ABO blood groups on the concentrations of FVIII and vWF in plasma. Research has shown that the FVIII-vWF complex directly impacts osteoclast activity, potentially playing a role in bone resorption by inducing cellular apoptosis. This process is closely linked to bone remodeling and potential bone loss. As a result, variations in FVIII and vWF levels among individuals may influence the changes observed in mandibular bone structure, potentially contributing to differences in mandibular asymmetry [15, 16].

ABO antigens are glycoproteins present in the body and possibly even in bone tissue, suggesting a biological link between ABO blood groups and osteoporosis [41]. In this context, studies have investigated the relationship between blood groups and bone mineralization or osteoporosis. It has been reported that individuals with non-O blood groups are at a higher risk for osteoporosis, particularly in individuals over the age of 50 and postmenopausal women [13].

Choi and Pai found that the prevalence of osteoporosis in the proximal femur and lumbar spine was 2.3 to 7.7 times higher in postmenopausal women with the AB blood group compared to those with blood group O [14]. In another study, women with blood group O were found to have significantly higher bone mineral density in the lumbar spine and proximal femur [13, 14, 41].

TMDs have a multifactorial etiology, including psychological components. Psychological studies have shown that TMD patients exhibit psychological dysfunctions similar to those seen in other chronic musculoskeletal pain disorders [42]. Some studies have reported a higher prevalence of psychiatric disorders in individuals with the AB blood group. Additionally, nitric oxide metabolism differences have been suggested as a possible factor influencing neurotransmitter activity. However, the direct relevance of these findings to mandibular asymmetry remains unclear and requires further investigation [43]. The observed higher condylar asymmetry in individuals with AB blood type raises questions about potential underlying factors. While some studies suggest a link between ABO blood groups and psychological traits, this relationship remains unclear. Future studies incorporating psychological assessments may help clarify this association.

A clinical cross-sectional study reported higher preoperative anxiety levels in individuals with the AB blood group compared to other blood groups [44]. The authors attributed the results to the role of glycosyltransferases, which catalyze ABO blood group antigens. Different glycosyltransferase activities are observed in different blood groups, and these enzymes play a role in the neuroinflammatory response. They are also reported to be important in nerve injury repair and regeneration through the glycosylation of glycoproteins in Schwann cells.

There are some limitations to consider when interpreting the findings of this study. Firstly, panoramic radiographs, which are commonly used for the assessment of mandibular asymmetry, are inherently prone to distortion and magnification errors. Although standard imaging protocols, head positioning techniques, and digital measurement tools were used to minimize these errors, cone beam computed tomography (CBCT) can more accurately assess mandibular asymmetry in three dimensions. Secondly, this study was conducted in a single population group and with a relatively limited sample, which may limit the generalizability of the findings to larger populations. Finally, the cross-sectional design of this study prevents us from establishing a causal relationship between ABO blood groups and mandibular asymmetry. Prospective studies examining blood group variations and bone metabolism markers, skeletal growth patterns, and histological changes may provide a better understanding of the mechanisms underlying this relationship.

The strengths of our study include the similarity of gender and age distributions of patient blood groups, the fact that our study is a subject that has never been addressed in the literature before, the measurement of condylar and ramal asymmetries by software and calibration, and the selection of patients free of factors that may affect mandibular asymmetry. Patients with known TMJ disorders were excluded to minimize potential confounding effects, as TMJ pathologies can directly impact condylar and ramal asymmetry. This approach ensured that the study focused on inherent skeletal variations rather than secondary changes due to joint disorders. However, our study was conducted on panoramic images and with a relatively limited sample size. Similar detailed studies on the subject are needed in more crowded populations.

## CONCLUSION

Due to the lack of previous studies investigating condylar and ramal asymmetries across different blood groups, comparisons of our results were not possible. Overall, these results provide important insights into the relationship between blood groups and asymmetry indices in a population without TMJ complaints, history of systemic diseases and orthodontic treatment. The statistically significant differences observed between AB and B blood groups in relation to condylar asymmetry indicate a potential association between blood type and mandibular anatomical variations; however, a causal relationship cannot be inferred from this study. The study findings also highlight the complexity of maxillofacial asymmetry, which may be influenced by a combination of genetic and environmental factors. Considering the multifactorial nature of craniofacial growth and development, it is essential to explore additional biological and biomechanical determinants that may contribute to these anatomical differences. Further studies are needed to confirm these findings and determine the mechanism underlying the association between blood group and mandibular asymmetry. Future research should also incorporate three-dimensional imaging techniques, such as CBCT, to provide more precise measurements and enhance the understanding of skeletal asymmetries in relation to blood type. Additionally, expanding the sample size to include diverse populations may help establish more generalizable conclusions regarding this potential relationship.

## Figures and Tables

**Fig. (1):**
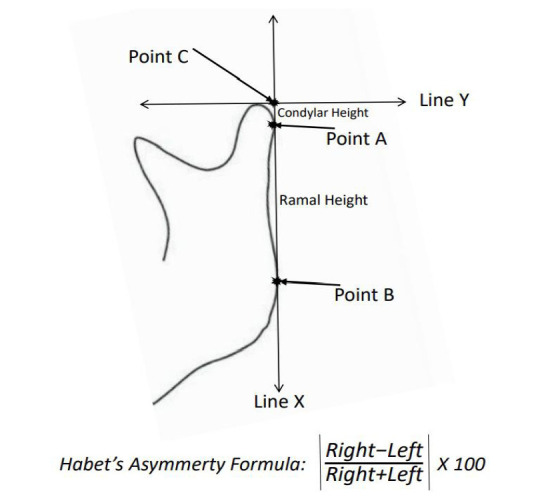
Habets formula for asymmetry.

**Fig. (2):**
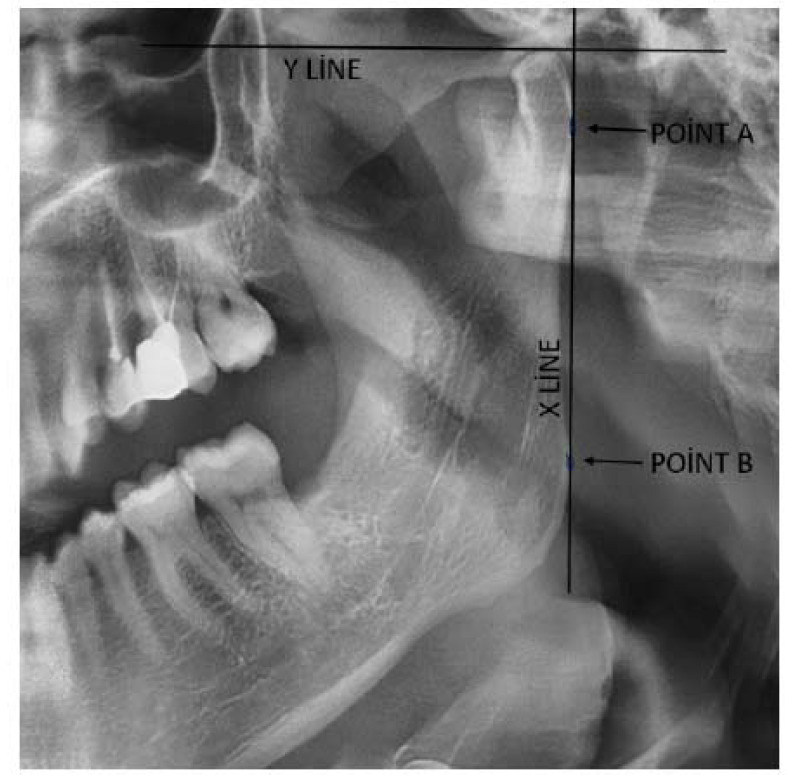
A panoramic drawing for Habets formula calculation.

**Table 1:** Inclusion and exclusion criteria.

**Inclusion Criteria**	**Exclusion Criteria**
Age ≥ 18 years	Age < 18 years
Suitable panoramic film quality (for asymmetry index calculation)	Unsuitable panoramic film quality (for asymmetry index calculation)
Complete demographic information (age, gender, blood type)	Incomplete demographic information (age, gender, blood type)
Absence of systemic diseases	Presence of systemic diseases (e.g., diabetes, autoimmune diseases).
Absence of craniofacial deformity	Presence of craniofacial deformity (congenital or acquired)
No orthodontic treatment history	History of orthodontic treatment or current orthodontic appliances
TMJ-related complaint absence	TMJ-related complaint presence
?	Inconsistent or incomplete patient records

**Table 2:** Statistical analysis for ABO groups.

**ABO Groups**									
	**A**		**B**		**AB**		**O**		**P value**
Sex distribution	Male	Female	Male	Female	Male	Female	Male	Female	,613 a
	26	28	12	11	6	12	23	31	
Age distribution	26,12±7.38		26,69±7,81		31,33±11,66		28,12±7,32		,148 b
Condylar asymmetry score	9,82±11,86 x y		7,33±6,46 y		13,60±9,79 x		9,40±9,95 x y		**,044 c**
Ramal asymmetry score	5,45±13,51		3,15±3,00		3,45±2,35		2,79±3,51		,344 b

a) Chi-square test. b) Kruskal Wallis test. c) Kruskal Wallis test with pairwise comparison including Bonferroni correction.

**Table 3:** Statistical analysis for RH groups.

**RH Groups**					
	**Rh +**		**Rh -**		**P value**
Sex distribution	Male	Female	Male	Female	,744 a
	63	76	4	6	
Age distribution	27,81±8,13		30,30±7,97		,109 b
Condylar asymmetry score	9,99±10,56		6,18±3,78		,371 b
Ramal asymmetry score	3,97±8,84		2,79±2,16		,952 b

a) Chi-square test. b) Mann Whitney U test.

## Data Availability

The data of current study are available from corresponding author, [M.P], on a reasonable request.

## LIST OF ABBREVIATIONS

TMDs: Temporomandibular Disorders
RH: Rhesus
RBCS: Red Blood Cells
TMJ: Temporomandibular Joint
CH: Condyle Height
RH: Ramal Height
CAI: Condylar Asymmetry Index
RAI: Ramal Asymmetry Index
FVIII: Factor VIII
vWF: von Willebrand factor

## STANDARDS OF REPORTING

STROBE guidelines were followed.
